# Opportunities of combinatorial thin film materials design for the sustainable development of magnesium-based alloys

**DOI:** 10.1038/s41598-021-97036-6

**Published:** 2021-08-31

**Authors:** Marcus Hans, Philipp Keuter, Aparna Saksena, Janis A. Sälker, Markus Momma, Hauke Springer, Jakub Nowak, Daniela Zander, Daniel Primetzhofer, Jochen M. Schneider

**Affiliations:** 1 Materials Chemistry, RWTH Aachen University, Aachen, Germany; 2 Metallic Composite Materials, RWTH Aachen University, Aachen, Germany; 3 Chair of Corrosion and Corrosion Protection, RWTH Aachen University, Aachen, Germany; 4 Department of Physics and Astronomy, Uppsala University, Uppsala, Sweden

**Keywords:** Metals and alloys, Surface chemistry

## Abstract

Magnesium-based lightweight structural materials exhibit potential for energy savings. However, the state-of-the-art quest for novel compositions with improved properties through conventional bulk metallurgy is time, energy, and material intensive. Here, the opportunities provided by combinatorial thin film materials design for the sustainable development of magnesium alloys are evaluated. To characterise the impurity level of (Mg,Ca) solid solution thin films within grains and grain boundaries, scanning transmission electron microscopy and atom probe tomography are correlatively employed. It is demonstrated that control of the microstructure enables impurity levels similar to bulk-processed alloys. In order to substantially reduce time, energy, and material requirements for the sustainable development of magnesium alloys, we propose a three-stage materials design strategy: (1) Efficient and systematic investigation of composition-dependent phase formation by combinatorial film growth. (2) Correlation of microstructural features and mechanical properties for selected composition ranges by rapid alloy prototyping. (3) Establishment of synthesis–microstructure–property relationships by conventional bulk metallurgy.

## Introduction

As resources are limited, materials scientists must consider economical, ecological and ethical boundary conditions for materials application and the development of new materials in order to contribute to a more sustainable society^1^. One of the current challenges is lightweight structural materials design, such as in the automotive sector, and magnesium exhibits enormous potential for energy savings based on its low density and high specific strength^2^. However, hexagonal magnesium (space group *P6*_*3*_*/mmc*) is poorly deformable at room temperature because of a limited number of operative slip systems^3^. As the intrinsic brittleness of magnesium hinders the application in structural systems, enhancement of the ductility is achieved through alloying approaches, where prominently aluminium, calcium and zinc are considered^4^.

It has been shown that incorporation of 0.3 at% calcium in Mg–3Al–1Zn (at%) results in a high strain hardening capability which in turn reduces mechanical instabilities during plastic deformation and, therefore, improves the uniform ductility^5^. Moreover, doping of magnesium with 0.90 at% aluminium and 0.06 at% calcium led to a significant macroscopic effect on the ductility by activation of pyramidal in addition to basal slip^6^. Microstructural characterisation revealed that this ductility increase was not caused by texture engineering, nano-structuring, grain size reduction, twinning activation or second phase dispersions, but simply a solid solution effect which facilitates the activation of non-basal dislocation slip^6^. Besides ductility, necessary for the fabrication of components, high strength is required for the operation of magnesium alloys under mechanical loading. Magnesium alloys with 0.3 at% calcium and varying aluminium concentration from 0 to 1.0 at%, having been produced by induction melting and age hardening increased the alloy resistance towards plastic deformation by > 70% for 0.3 at% aluminium^7^. Plate-like precipitates were observed for the peak-aged sample and interpreted as ordered Guinier–Preston zones which contribute significantly to the strengthening of the alloy and similar findings were obtained for Mg–0.3Ca–0.3Zn (at%)^7,8^. In terms of chemical stability, Mg–9Al–0.5Ca (at%) was investigated by hydrogen collection and potentiodynamic corrosion testing; a reduction of the corrosion rate by > 80% in comparison to unmodified Mg–9Al (at%) was explained by microstructural refinement and consequently an increased fraction of intermetallic cubic Mg_17_Al_12_ (space group *I*$\overline{4 }$*3 m*) at the grain boundaries^9^.

From the discussion above it is evident that most of the magnesium alloy design studies are currently focused on a limited number of alloying elements and concentration ranges. The current conventional approach typically consists of several iterative loops including bulk casting of a single alloy composition (often in dimensions of several kilograms up to tons), thermomechanical treatment, specimen fabrication and various testing procedures. While this kind of incremental approach provides robust data and detailed insights into synthesis–microstructure–property relationships, it is time, energy, and material intensive. Bulk combinatorial techniques, referred to as rapid alloy prototyping, allow for substantial acceleration and increased efficiency via parallelisation and effective reduction as well as through-process streamlining^10^, but are still limited to investigations of the mechanical and constitutional profile of about 50 material states (i.e. alloy compositions and thermo-mechanical parameters) per week.

Thin film deposition techniques such as magnetron sputtering provide opportunities in this context since complex chemistries, including all metals and non-metals, are easily accessible by the design of the sputtering materials and the reactive gas atmosphere^11–13^. Moreover, a vast chemical composition space is accessible by combinatorial thin film deposition in which the geometric arrangement of the sputtering sources enables tailoring of a composition distribution on the substrate with more than 100 different individual chemical compositions being attainable per day within a single thin film growth experiment^14,15^. The lower boundary amounts for investigations into the composition-dependent phase formation of structural materials are on the order of ten grams for bulk processing and ten milligrams for thin films. Hence, given the same amount of material for both processing strategies, combinatorial thin film growth offers a factor of 10^5^ higher efficiency than conventional bulk processing. The combinatorial approach has been e.g. successfully applied to the design of ultra-strong metallic glasses or low-cost alternatives to nickel-base superalloys for medium-to-high temperature structural applications^16,17^.

Based on the previous paragraph, it is clear that combinatorial thin film materials design may provide opportunities for the sustainable screening of novel magnesium-based alloys by efficient and systematic investigation of the composition-dependent phase formation. However, a major limitation of thin film synthesis is the incorporation of impurities in situ (during deposition^18^) or ex situ (after exposure to ambient^19^). Since calcium readily reacts upon exposure to oxygen and water vapour^20,21^, the magnesium–calcium system was selected as “worst case” scenario for evaluation of the opportunities provided by thin film synthesis for the design of novel magnesium-based alloys. Thus, the present work is focused on the microstructural control of impurity incorporation in magnesium–calcium thin films while discussing the implications thereof for the sustainable development of novel magnesium-based alloys.

## Results

### Thin film growth and microstructure

**Figure 1:**
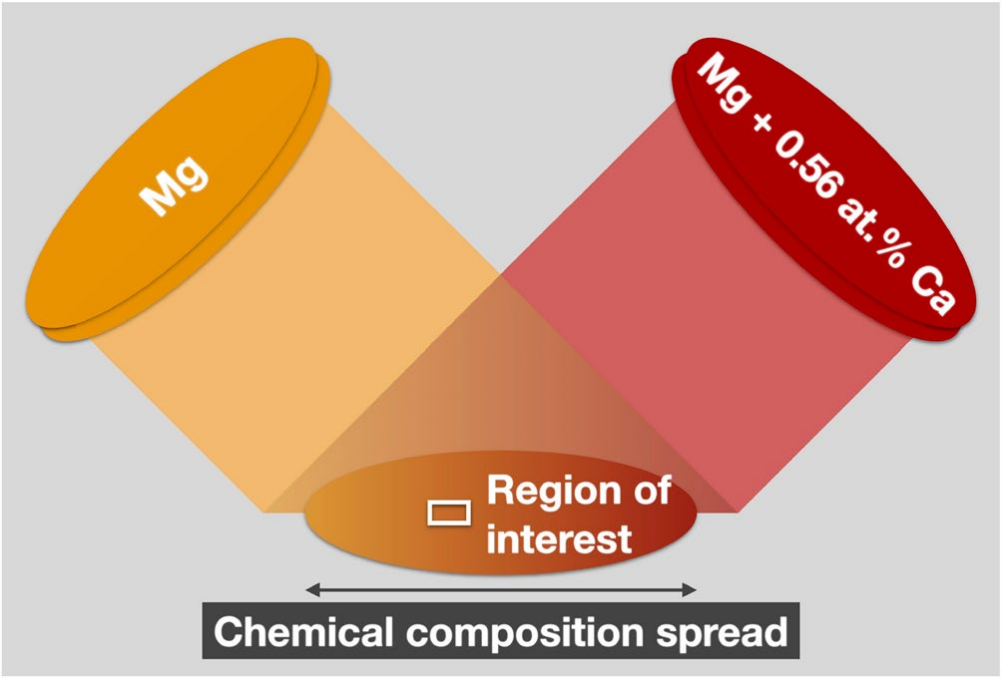
Combinatorial growth of (Mg,Ca) solid solutions with a chemical composition spread induced by the co-sputtering geometry. An elemental magnesium as well as a cast calcium-alloyed magnesium target with a calcium concentration of 0.56 at% were used. The chemical composition spread on the substrate is indicated by the colour gradient and the region of interest for microstructural as well as chemical composition analysis at the nanometre scale is indicated by the rectangular box.

(Mg,Ca) solid solution thin films with hexagonal crystal structure were grown by combinatorial magnetron sputtering (Fig. 1). The growth temperature was varied between homologous temperatures T* = *T*/*T*_melt_ of 0.32 (without intentional heating) and 0.40 (100 °C temperature), respectively, and a region with a calcium concentration < 0.4 at% was selected for further analysis since such alloying levels are expected to result in the formation of bulk single-phase solid solutions^4,5,7–9^.

**Figure 2:**
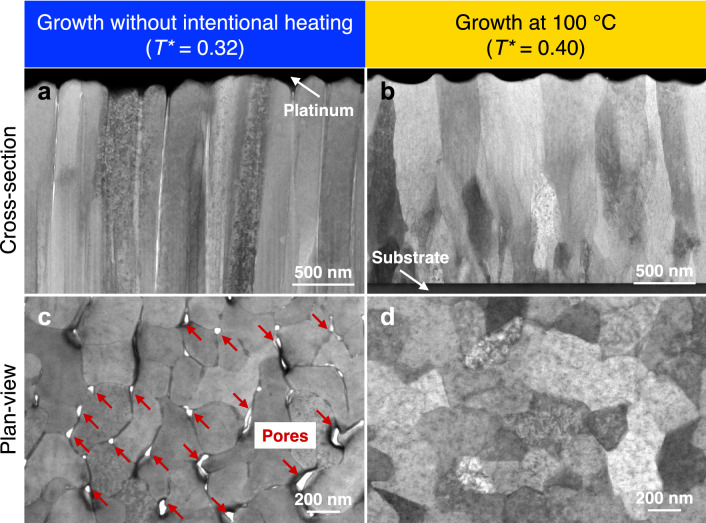
Microstructural characterisation of (Mg,Ca) solid solution thin films grown at different temperatures. Cross-section-views of the thin films (**a**) without intentional heating and (**b**) grown at 100 °C temperature. Plan-views of the thin films (**c**) without intentional heating and (**d**) grown at 100 °C temperature. All images were acquired in bright field mode and the formation of pores at the columnar grain boundaries is evident for T* = 0.32. Homologous temperatures T* = *T*/*T*_melt_ were calculated by using the melting temperature of magnesium *T*_melt_ = 923 K and assuming room temperature (298 K) for the growth scenario without intentional heating as a lower boundary. X-ray diffraction as well as transmission Kikuchi diffraction data can be found in Fig. SI1.

The consequences of growth temperature for the microstructure were evaluated by scanning transmission electron microscopy (Fig. 2). The film grown at T* = 0.32 exhibits an underdense, columnar microstructure and plan-view images emphasise the presence of open grain boundaries, in particular at the triple junctions (Fig. 2a,c). In contrast, significant densification was accomplished by employing a growth temperature of T* = 0.40 (Fig. 2b,d).

### Thin film chemical composition and impurity incorporation

**Figure 3:**
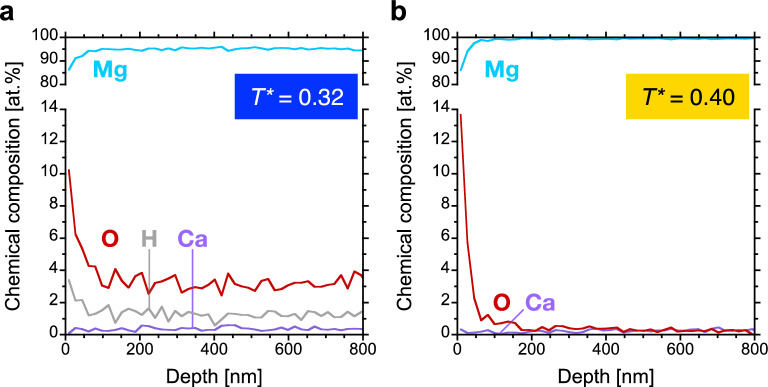
Thin film chemical compositions. Elastic recoil detection analysis depth profiles are presented for the (Mg,Ca) solid solution thin films grown at (**a**) T* = 0.32 and (**b**) T* = 0.40. In addition, average calcium concentrations were determined by Rutherford backscattering spectrometry and the corresponding data can be found in Fig. SI2. The figures were created with KaleidaGraph 4.5.3 (https://www.synergy.com).

Furthermore, the thin film chemical composition was examined by ion beam analysis using an iterative and self-consistent approach in order to enhance its accuracy^22^. While elastic and Rutherford backscattering are employed for thickness measurements and effective quantification of heavier constituents within a light matrix, respectively^23,24^, elastic recoil detection analysis provides accurate information on light species such as oxygen or hydrogen^23^. Depth profiles are shown in Fig. 3 and while surface oxidation is apparent for both thin films, a substantial reduction of the average oxygen concentration (determined for depths > 100 nm) from 3.2 to 0.35 at% is observed as the growth temperature is increased from T* = 0.32 to 0.40. The low oxygen signal of the thin film grown at T* = 0.40 contains approximately 25% background noise, and thus, the actual oxygen concentration is indeed < 0.3 at%. Moreover, an average hydrogen concentration of approximately 1 at% is detected for the microstructure with open grain boundaries (T* = 0.32), while the hydrogen content of the film with dense microstructure (T* = 0.40) is below the detection limit of 0.1–0.2 at%. Calcium concentrations of 0.37 (T* = 0.32) and 0.30 at% (T* = 0.40) were measured by Rutherford backscattering spectrometry (see Fig. SI2), showing that the obtained thin film densities of 1.71 ± 0.09 and 1.84 ± 0.10 g cm^−3^ (the density of an ideal Mg_99.6_Ca_0.4_ alloy is 1.74 g cm^−3^) emphasise a growth temperature-induced densification of 8%. This densification is in line with the structure zone diagram proposed by Thornton as the formation of underdense thin film morphologies is overcome by increasing the homologous temperature, thereby enabling a larger adatom mobility^25^. Hence, it is evident that the increase in growth temperature from T* = 0.32 to 0.40 significantly enhances the adatom mobility, resulting in grain boundary densification (Fig. 2) which concomitantly impedes oxygen and hydrogen incorporation into the thin films (Fig. 3).

### Nanometre scale chemical composition and impurity incorporation

In order to determine the local chemical composition of the grain boundaries, laser-assisted atom probe tomography was employed. This technique is based on the field evaporation of atoms by supplying a voltage of a few kilovolts to needle-shaped specimens with its radius at the apex on the order of tens of nanometres^26,27^. Subsequent to field evaporation, the atoms are ionised by the surface electric field at the specimen^28^. The combination of the voltage with laser pulses causes a temporal increase of the specimen temperature above the evaporation threshold, enabling a time-of-flight measurement for each evaporated atom and providing mass-to-charge state ratios. The original position of each atom in the needle-like specimen before evaporation can be reconstructed based on the data record from a position-sensitive detector. Thus, atom probe tomography is applied for spatially-resolved chemical composition characterisation at the nanometre scale^29^ and therefore ideally suited for the quantitative probing of grain boundaries.

**Figure 4:**
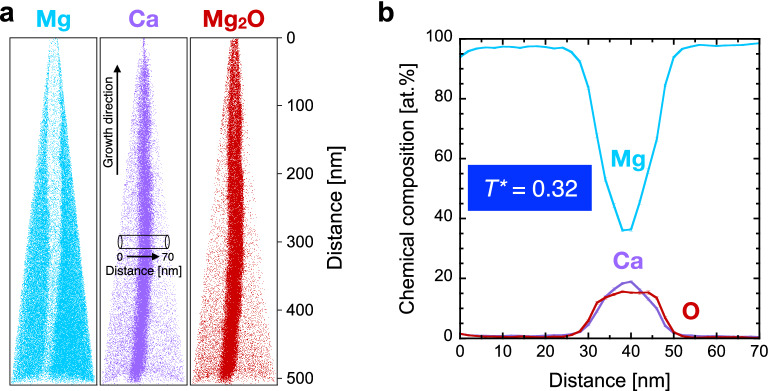
Three-dimensional chemical composition characterisation at the nanometre scale of the (Mg,Ca) solid solution thin film grown at T* = 0.32. (**a**) Reconstruction of detected Mg and Ca ions as well as Mg_2_O molecular ions. The cylinder with a length of 70 nm indicates the region from the chemical composition profile which is presented in (**b**). The corresponding mass spectrum and applied ranging as well as atomic positions of O can be found Fig. SI3, Table SI1 and Fig. SI4, respectively. Figure (**a**) was created with IVAS 3.8.0 (https://www.atomprobe.com) and Fig. (**b**) was created with KaleidaGraph 4.5.3 (https://www.synergy.com).

The reconstruction of an atom probe specimen from the (Mg,Ca) solid solution thin film grown at T* = 0.32 with atomic positions of detected Mg and Ca ions as well as Mg_2_O molecular ions provides evidence for the presence of a grain boundary (Fig. 4a). The grain boundary diameter is on the order of 20 nm and in agreement with the previous microstructural characterisation (Fig. 2a,c). The chemical composition profile (Fig. 4b) reveals the segregation of up to 19 at% calcium to the grain boundary, while the calcium concentration is < 1 at% within the two adjacent grains in agreement with the solid solubility limit of calcium in magnesium^30^. It is evident that the open grain boundaries (Fig. 2a,c) facilitate ex situ oxygen incorporation of up to 16 at% due to atmosphere exposure (Fig. 4).

**Figure 5:**
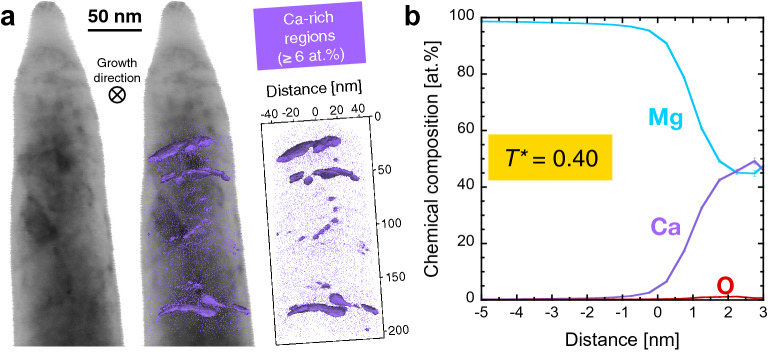
Correlative microstructural and compositional characterisation of the (Mg,Ca) solid solution thin film grown at T* = 0.40, combining scanning transmission electron microscopy and atom probe tomography. (**a**) Bright field image of an atom probe specimen, reconstruction of calcium atomic positions with highlighted calcium-rich regions (≥ 6 at%) as well as an overlay of the bright field image and the atom probe reconstruction. (**b**) Proximity histogram which reveals the evolution of chemical composition from the grains into the grain boundaries. 0 nm distance corresponds to the threshold composition of 6 at% calcium as indicated in (**a**). The corresponding mass spectrum and applied ranging as well as atomic positions of Mg, Ca, O and Mg_2_O can be found in Fig. SI5, Table SI2 and Fig. SI4, respectively. Figure (**a**) was created with IVAS 3.8.0 (https://www.atomprobe.com) and Fig. (**b**) was created with KaleidaGraph 4.5.3 (https://www.synergy.com).

While the presence of a grain boundary is obvious in case of the atom probe dataset for the thin film with open grain boundaries (T* = 0.32), unambiguous identification of grain boundary regions in the dense (Mg,Ca) solid solution (T* = 0.40) by atom probe tomography appears challenging since the structural information in such datasets is limited^31^. Efficient correlation of microstructure and local chemical composition was realised by employing scanning transmission electron microscopy on an atom probe specimen of the (Mg,Ca) solid solution thin film with dense microstructure, grown at T* = 0.40 (Fig. 5). The scanning transmission electron microscopy image reveals contrast differences with dark lines corresponding to grain boundaries (compare Fig. 2d). The reconstruction after the atom probe measurement shows the atomic positions of calcium and the overlay image emphasises that the calcium-rich regions coincide with grain boundaries (Fig. 5a). It should be noticed that the detection efficiency of the atom probe microscope was 36% and atoms from the boundaries of the analysed volume may be lost because of aberrations of the ion trajectories during flight towards the detector^32^. Therefore, not all of the grain boundaries visible by scanning transmission electron microscopy at the surface of the atom probe specimen are represented in the reconstruction. The proximity histogram (Fig. 5b) reveals the chemical composition evolution from the grain interior into the boundaries. Also, for the growth temperature of T* = 0.40, strong preferential segregation of calcium to the grain boundaries is apparent by an increase in the calcium amount from < 1 up to 49 at%. However, while an ex situ oxygen incorporation of up to 16 at% was identified for T* = 0.32 (Fig. 4), maximum oxygen impurities are only 1 at% at the grain boundaries for T* = 0.40 and < 0.2 at% within the grains (Fig. 5), in excellent agreement with ion beam analysis data (Fig. 3).

### Bulk alloy impurity incorporation

**Figure 6:**
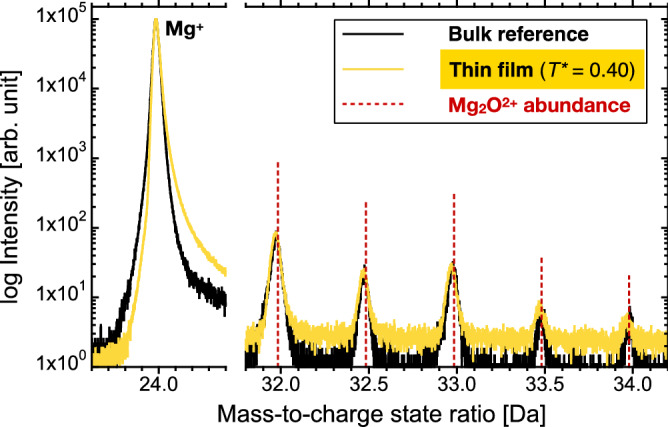
Mass spectrum data of the (Mg,Ca) solid solution grown at T* = 0.40 in comparison to a Mg–1.06Al–0.046Ca (wt%) bulk solid solution. Both mass spectra were normalised with respect to the maximum intensity of 1 × 10^5^ for the ^24^Mg^+^ peak. Dashed lines represent the abundance of the Mg_2_O^2+^ isotopes. The figure was created with KaleidaGraph 4.5.3 (https://www.synergy.com).

Oxygen impurity levels of bulk structural materials are commonly analysed by infrared absorption spectroscopy of a melt in a graphite crucible. However, this technique is challenging for magnesium-based materials with respect to oxygen impurity quantification since the magnesium vapour recombines with carbon monoxide or dioxide and condenses as MgO^33^. In order to compare the oxygen impurity level of thin films to bulk-processed alloys, the chemical composition within a grain of a cast Mg–1.06Al–0.046Ca (wt%) solid solution was investigated by atom probe tomography. The impurity levels of the thin film and the bulk alloy are emphasised by mass spectrum data (Fig. 6). Both mass spectra were normalised to the most abundant species of ^24^Mg^+^ and the amount of oxygen impurities is governed by detected Mg_2_O^2+^ molecular ions (see Figs. SI3, SI4, SI5 and SI6). It is clear that the amount of detected Mg_2_O^2+^ is very similar for the bulk alloy as well as the thin film grown at T* = 0.40. The minimum amount of oxygen in the bulk alloy was 0.2 at% and therefore on the same level as in the thin film grains grown at T* = 0.40 with < 0.2 at%.

## Discussion

(Mg,Ca) solid solutions with an average calcium concentration of < 0.4 at% were selected from combinatorial thin film deposition experiments. The microstructure-dependent impurity incorporation in magnesium–calcium thin films was evaluated by combination of scanning transmission electron microscopy, ion beam analysis and atom probe tomography data. In case of T* = 0.32 an underdense microstructure was formed with open columnar grain boundaries which are shown to facilitate significant oxygen incorporation up to 16 at% due to atmosphere exposure. Increasing the growth temperature to T* = 0.40 enhanced adatom mobility and thereby caused grain boundary densification. Consequently, oxygen incorporation is limited to 1 at% at the grain boundaries and to < 0.2 at% within the grains. The minimum amount of oxygen measured in a grain of a reference bulk alloy was 0.2 at% and therefore on the same level as the thin film grown at T* = 0.40 with < 0.2 at%.

Since impurity incorporation of the thin films is very similar to bulk magnesium alloys, control of the microstructure positions thin film growth as efficient screening opportunity to exploit large chemical composition spaces within a single synthesis experiment for the design of magnesium-based alloys. The combinatorial thin film growth approach enables the efficient and systematic investigation of composition-dependent phase formation for more than 100 individual chemical compositions per growth experiment. Subsequently, a selected composition range can be transferred to rapid alloy prototyping techniques for the initial correlation of microstructural features and macroscopic mechanical properties^10^. Finally, upscaling to conventional bulk metallurgy will provide robust data in order to establish synthesis–microstructure–property relationships.

In order to substantially reduce time, energy, and material requirements for the sustainable development of novel magnesium-based alloys with improved properties, we propose a three-stage materials design strategy:

1. Efficient and systematic investigation of the composition-dependent phase formation by combinatorial thin film growth.
2. Correlation of microstructural features and macroscopic mechanical properties for a selected chemical composition range by rapid alloy prototyping.
3. Establishment of synthesis–microstructure–property relationships based on conventional bulk metallurgy.

We envision that future design efforts to other structural and functional materials can be carried out sustainably by adopting the here communicated synthesis strategy.

## Methods

### Thin film synthesis

(Mg,Ca) solid solution thin films were deposited by direct-current magnetron sputtering in a high vacuum chamber, evacuated to a base pressure of 3 × 10^–5^ Pa. One elemental magnesium and one cast calcium-alloyed magnesium target, with a calcium concentration of 0.56 at% (based on wet chemical analysis) were used. The raw materials magnesium and calcium for the (Mg,Ca) target exhibited a purity of > 99.95 and > 98.8 (wt%) respectively. Both circular targets were machined to a diameter of 50 mm. The targets were positioned on cathodes at an inclination angle of 45° relative to the substrate normal at a target-to-substrate distance of 10 cm and both cathodes were powered with 50 W. Depositions were carried out without intentional heating (T* = 0.32) for 60 min and at a substrate temperature of 100 °C (T* = 0.40) for 30 min, resulting in thin films of approximately 4.0 and 1.6 μm thickness, respectively. The argon (99.999% purity) partial pressure was kept constant at 0.5 Pa and silicon (100) substrates were employed.

### Bulk synthesis

A charge of approximately 200 g of technically pure elements (Mg–1.06Al–0.046Ca in wt%, purity of Al > 99.999 wt%) were inductively molten in a steel crucible under an argon atmosphere of approximately 10 bar absolute pressure), and cast into a copper mould with the internal dimensions of 30 × 60 mm^2^. The surfaces of the cast block were milled, the block was annealed at 450 °C in air for 20 min and directly hot rolled to 50% total reduction (with about 10% reduction per pass and reheating for approximately 10 min). After the last rolling pass, the block was reheated to 450 °C for 15 min and quenched in water to room temperature. A sample with the dimensions of 15 × 20 × 5 mm was taken from the centre of the rolled material perpendicular to the rolling direction by wet cutting, using a corundum blade. Manual grinding was carried out, using SiC abrasive paper with grit sizes of 500 and 1000 in water as well as with grit sizes of 2500 and 4000 in 99.8% ethanol, while applying minimal pressure. Subsequently, the sample was polished using anhydrous diamond suspensions of 3, 1 and 0.25 μm as well as anhydrous Struers DP-Lubricant Blue on polishing cloths. Between each preparation step a one-minute ultrasonic cleaning in 99.8% ethanol was performed.

### Ion beam analysis

All ion beam-based composition depth profiling was performed using the 5MV 15-SDH-2 pelletron tandem accelerator at Uppsala University^34^. Rutherford backscattering spectrometry and elastic backscattering spectrometry were done using a beam of 2 MeV and 3.5 MeV He^+^ primary ions. Scattered ions were detected using a semiconductor detector positioned at a backscattering angle of 170 degrees with respect to the primary beam. Backscattering spectra were analysed using the SIMNRA simulation package^35^. Time-of-flight elastic recoil detection analysis was performed using a primary beam of 36 MeV I^8+^ ions. Recoils were detected employing a combined time-of-flight and energy detection telescope system as described in ref*.*^36^ positioned at a 45° deflection angle with respect to the primary beam. Composition depth profiles were extracted using the CONTES package^37^. The combination of backscattering spectrometry and recoil detection analysis significantly reduces systematic uncertainties as discussed in ref.^38^.

### Scanning transmission electron microscopy

Thin lamellae were prepared by focused ion beam techniques with a FEI Helios Nanolab 660 dual-beam microscope using gallium ions at 30 kV. Cross-sectional as well as plan-view lamellae were prepared and the latter were extracted from thin film cross-sections at thicknesses of approximately 2 μm (T* = 0.32) and 1 μm (T* = 0.40). Final thinning to approximately 100 nm lamella thickness was performed by using a 30 kV voltage and 80 pA current. Scanning transmission electron micrographs from lamellae as well as atom probe specimens (see Fig. SI7) were acquired in bright field mode at 30 kV and 50 pA using a STEM III detector.

### Atom probe tomography

Atom probe specimens were prepared by focused ion beam techniques following a standard recipe^39^. Specimen cleaning was done at 5 kV voltage of the focused ion beam using a current of 40 pA for 30 to 60 s. Quantitative probing of the chemical composition at the nanometre scale was carried out with a CAMECA locale electrode atom probe 4000X HR in laser-assisted measurement mode. Specimens from thin films grown at T* = 0.32 were evaporated with 30 pJ laser pulse energy, 250 kHz laser pulse frequency, 60 K base temperature and 0.5% detection rate (dataset shown in Fig. 4). Specimens from thin films grown at T* = 0.40 as well as the bulk reference were evaporated with different parameters (see Fig. SI8) of 100 pJ, 125 kHz, 30 K and 0.5% (datasets shown in Figs. 5 and 6). Data analysis was done with the IVAS 3.8.0 software and the shank angle protocol was used for the reconstructions.

## Supplementary Information

Supplementary Information.

## Data Availability

The authors declare that all relevant data supporting the findings of this study are available within the article and its supplementary information.
