# Supplementary material for: Opportunities of combinatorial thin film materials design for the sustainable development of magnesium-based alloys
Source: Sci Rep. 2021 Aug 31;11:17454. doi: 10.1038/s41598-021-97036-6 (PMC8408169; doi:10.1038/s41598-021-97036-6)
Supplement: Supplementary file 1 — Supplementary Information. [file 41598_2021_97036_MOESM1_ESM.docx]

**Supplementary information**

**Opportunities of combinatorial thin film materials design for the sustainable development of magnesium-based alloys**

Marcus Hans^1^*, Philipp Keuter^1^, Aparna Saksena^1^, Janis A. Sälker^1^, Markus Momma^1^, Hauke Springer^2^, Jakub Nowak^3^, Daniela Zander^3^, Daniel Primetzhofer^4^, Jochen M. Schneider^1^

^1^ Materials Chemistry, RWTH Aachen University, Aachen, Germany.

*email: hans@mch.rwth-aachen.de

^2^ Metallic Composite Materials, RWTH Aachen University, Aachen, Germany

^3^ Chair of Corrosion and Corrosion Protection, RWTH Aachen University, Aachen, Germany

^4^ Department of Physics and Astronomy, Uppsala University, Uppsala, Sweden

**Table of contents**

[Phase formation and grain orientation 2](#_Toc79060316)

[Quantification of calcium by ion beam analysis 4](#_Toc79060317)

[Ranging of atom probe mass spectra 5](#_Toc79060318)

[Detection of Mg_2_O molecular ions 10](#_Toc79060319)

[Scanning transmission electron microscopy of atom probe specimens 11](#_Toc79060320)

[References 13](#_Toc79060321)

# Phase formation and grain orientation

X-ray diffraction experiments were conducted in a Siemens D5000 diffractometer operated at a voltage of 40 kV and a current of 40 mA using Cu K_α_ radiation. Bragg-Brentano scans in a 2*θ* range of 10 to 90° in unlocked coupled mode with an offset of 2° were performed to avoid diffraction signals from the single crystal silicon (100) substrate. The step size was 0.05°.

Transmission Kikuchi diffraction was employed on plan-view thin lamellae in regions of 1×1 μm using a Hicari camera with a forward-scatter detector. The acceleration voltage and current were 30 kV and 1.6 nA, respectively, and the step size was 20 nm.

**Fig. SI1.** Phase formation and grain orientation. (**a**), Diffractogram and (**b**), transmission Kikuchi diffraction of the thin film grown at *T** = 0.32. (**c**), Diffractogram and (**d**), transmission Kikuchi diffraction of the thin film grown at *T** = 0.40. Diffraction reference lines of hexagonal magnesium are from the International Centre for Diffraction Data (00-035-0821). Fig. SI1a and c were created with KaleidaGraph 4.5.3 (https://www.synergy.com). Fig. SI1b and were created with OIM Analysis 8.5.0 (https://www.edax.com/).

The phase formation and grain orientation of the (Mg,Ca) solid solutions is shown in Fig. SI1. For both diffractograms (Fig. SI1a and c), all peaks correspond to a hexagonal magnesium crystal structure and the broad hump in the 2*θ* range from 60 to 80° originates from the silicon substrate. Enhancing the adatom mobility from *T** = 0.32 to 0.40 causes a change in the texture of the hexagonal grains from mixed (0002)/(10$\bar{1}$2)/(10$\bar{1}$3) towards the close-packed and therefore energetically-preferred (0002) orientation. This change in texture is also visible in the transmission Kikuchi diffraction data from plan-view lamellae (Fig. SI1b and d).

Average grain sizes are 263 ± 32 nm (*T** = 0.32) and 279 ± 39 nm (*T** = 0.40), thus, similar for both growth temperatures which can be rationalised by the fact that the formation of larger grains requires the activation of bulk diffusion at even higher temperatures of *T** > 0.7.^1^ However, the accessible growth temperature range is limited for magnesium-based films since thermally-induced desorption of magnesium has been reported recently for Mg_2_Ca Laves phase thin films grown at temperatures ≥ 150 °C (*T** ≥ 0.43)^2^ and similar effects were observed for the growth of (Mg,Ca) solid solutions.

# Quantification of calcium by ion beam analysis

Rutherford backscattering spectra (Fig. SI2) allow for accurate quantification of the calcium concentration. For assessing the density of the films, additional spectra were recorded at 3.5 MeV primary energy to obtain the total areal densities of the films, using ERDA depth profiles for light elements as additional input. The accuracy of the resulting densities is limited to ± 5% by the accuracy of the stopping power for the present compound. The relative comparison, however, is possible with significantly higher accuracy. During all backscattering experiments, the yields of X-rays emitted from the samples during irradiation (particle induced X-ray emission) were also recorded. The corresponding particle induced X-ray yields (not shown) permit to unambiguously identify the heavy constituent as calcium and show that the concentrations of argon (which could be incorporated from the working gas during sputtering) are below 0.01%.

**Fig. SI2.** Rutherford backscattering spectra from the thin films grown at *T** = 0.32 and 0.40, recorded using a primary beam of 2 MeV He^+^ ions. The figure was created with KaleidaGraph 4.5.3 (https://www.synergy.com).

# Ranging of atom probe mass spectra

Ranging of atom probe mass spectrum data is a crucial step since it determines the chemical nature of each reconstructed atom. All peaks of the mass spectrum have to be assigned to chemical species with suitable mass-to-charge state ratio, e.g. single and double ionised magnesium isotopes are expected at mass-to-charge state ratios of 24, 25 and 26 as well as 12, 12.5 and 13 Da, respectively. The mass spectrum of the reconstructions in Fig. 4 (*T** = 0.32) and Fig. 5 (*T** = 0.40) are shown below together with a detailed list of assigned peaks (Fig. SI3, Table SI1 and Fig. SI5, Table SI2, respectively). Moreover, reconstructions of detected Mg, Ca and O ions as well as Mg_2_O molecular ions are compared for both (Mg,Ca) solid solution thin films grown at *T** = 0.32 and 0.40 (Fig. SI4).

**Fig. SI3.** Mass spectrum of the atom probe specimen from the thin film grown at *T** = 0.32 (see Fig. 4). The figure was created with KaleidaGraph 4.5.3 (https://www.synergy.com).

**Table SI1.** Assignment of peaks from the mass spectrum (*T** = 0.32).

| Mass-to-charge state ratio [Da] | Detected ion^charge state^ |
| --- | --- |
| 12.0, 12.5, 13.0 | Mg^2+^ |
| 13.5 | Al^2+^ |
| 14.0, 15.0 | N^+^ |
| 16.0 | O^+^ |
| 17.0 | OH^+^ |
| 18.0 | OH_2_^+^ |
| 19.0 | OH_3_^+^ |
| 20.0 | Ca^2+^ |
| 20.5 | CaH^2+^ |
| 21.0, 21.5, 22.0 | MgOH_2_^2+^ |
| 24.0, 25.0, 26.0 | Mg^+^ |
| 24.5 | Mg_2_^+^ |
| 27.0 | Al^+^ |
| 28.0 | CaO^2+^ |
| 29.0 | CaOH_2_^2+^ |
| 30.0, 31.0 | Mg_2_C^2+^ |
| 32.0, 32.5, 33.0, 33.5, 34.0 | Mg_2_O^2+^ |
| 40.0, 44.0 | Ca^+^ |
| 40.5 | Ca_2_H^2+^ |
| 41.0, 45.0 | CaH^+^ |
| 41.5 | Ca_2_H_3_^2+^ |
| 42.0 | CaH_2_^+^ |
| 43.0 | CaH_3_^+^ |
| 56.0 | MgO_2_^+^ |
| 57.0 | MgO_2_H^+^ |
| 58.0 | MgO_2_H_2_^+^ |
| 59.0, 60.0, 61.0 | MgO_2_H_3_^+^ |
| 69.0 | Ga^+^ |
| 80.0 | Mg_2_O_2_^+^ |
| 81.0 | Mg_2_O_2_H^+^ |
| 82.0 | Mg_2_O_2_H_2_^+^ |

**Fig. SI4.** Reconstruction of detected Mg, Ca and O ions as well as Mg_2_O molecular ions of the (Mg,Ca) solid solution thin films grown at *T** = 0.32 and 0.40. The figure was created with IVAS 3.8.0 (https://www.atomprobe.com).

**Fig. SI5.** Mass spectrum of the atom probe specimen from the thin film grown at *T** = 0.40 (see Fig. 5). The figure was created with KaleidaGraph 4.5.3 (https://www.synergy.com).

**Table SI2.** Assignment of peaks from the mass spectrum (*T** = 0.40).

| Mass-to-charge state ratio [Da] | Detected ion^charge state^ |
| --- | --- |
| 12.0, 12.5, 13.0 | Mg^2+^ |
| 16.0 | O^+^ |
| 20.0, 21.0, 22.0 | Ca^2+^ |
| 24.0, 25.0, 26.0 | Mg^+^ |
| 24.5, 25.5 | Mg_2_^+^ |
| 27.0 | Al^+^ |
| 28.0 | CaO^2+^ |
| 32.0, 32.5, 33.0, 33.5, 34.0 | Mg_2_O^2+^ |
| 40.0 | Ca^+^ |
| 40.5 | Mg_2_O_2_^2+^ |
| 41.0, 43.0 | CaH^+^ |
| 41.5 | Ca_2_H_3_^2+^ |
| 42.0 | CaH_2_^+^ |
| 44.0, 44.5, 45.0, 45.5, 46.0 | Mg_2_Ca^2+^ |
| 48.0, 49.0, 50.0, 51.0 | Mg_2_^+^ |
| 52.0, 52.5, 53.0, 53.5, 54.0, 54.5 | Mg_2_CaO^2+^ |
| 55.0 | Al_2_CaO^2+^ |
| 56.0 | MgO_2_^+^ |
| 59.0 | MgO_2_H_3_^+^ |
| 64.0, 65.0, 66.0 | MgCa^+^ |
| 69.0 | Ga^+^ |
| 80.0, 81.0, 82.0 | Mg_2_O_2_^+^ |
| 93.0, 94.0, 95.0 | MgGa^+^ |

# Detection of Mg_2_O molecular ions

It is evident from the mass spectrum data as well as the reconstructions that most species contain magnesium, calcium and oxygen. Most critical is the potential overlap of peaks from Mg_2_O^2+^ as well as MgCa^2+^ and it seems unclear which of these species dominate the peaks in the mass-to-charge state ratio range of 32 to 34 Da. Therefore, a comparison between mass spectrum data of the (Mg,Ca) solid solution grown at *T** = 0.40 and a Mg/Al multilayer thin film was used (Fig. SI6). The Mg/Al multilayer thin film does not contain calcium and besides a higher level of background counts, the abundance of the isotopes is very similar to the abundances of the (Mg,Ca) solid solution. Therefore, the peaks in the mass-to-charge state ratio range from 32 to 34 Da were entirely assigned to Mg_2_O^2+^ ions. It is evident that the oxygen composition, quantified by atom probe tomography, represents an upper boundary since the detection signal may partially originate from MgCa^2+^ ions.

**Fig. SI6.** Comparison of mass spectrum abundances from the (Mg,Ca) solid solution (*T** = 0.40) as well as a Mg/Al multilayer thin film in the mass-to-charge state ratio range from 32 to 34 Da. The figure was created with KaleidaGraph 4.5.3 (https://www.synergy.com).

# Scanning transmission electron microscopy of atom probe specimens

Recent advances are correlative techniques and the combination of transmission electron microscopy and atom probe tomography enables the identification of chemical and structural states confined at dislocations or grain boundaries^3-5^. However, this approach is expensive as the needle-shaped, nanometre-sized atom probe specimens have to survive the handling and ambient exposure after preparation during transport to the transmission electron as well as to the atom probe microscope. In addition, extensive characterisation by electrons accelerated to several hundred keV may cause beam damage to the specimen. In order to efficiently correlate microstructure and local chemical composition at the nanometre scale, a different strategy was employed (Fig. SI7). Atom probe specimens are commonly prepared in commercial dual-beam microscopes^6^ and these platforms can be equipped with scanning transmission electron microscopy detectors for structural characterisation using electrons at 30 kV energy. A holder was designed which allows for microstructural characterisation of the atom probe specimens in transmission geometry directly after preparation and using the identical dual-beam microscope platform. Subsequent to the microstructural characterisation, the specimens were transferred to the atom probe microscope with a total ambient exposure time < 5 minutes which minimises additional oxygen uptake.

**Fig. SI7.** Setup for structural characterisation by scanning transmission electron microscopy (STEM) of atom probe specimens. (**a**, **b**), Design of sample holder to load the atom probe specimens, located on a silicon coupon which is fixed on a copper clip, in transmission geometry. (**c**), Electron microscopy image of the atom probe specimens. (**d**), Immediate transfer of the atom probe specimens after scanning transmission electron microscopy characterisation to a CAMECA local electrode atom probe 4000X HR (view inside buffer chamber for specimen storage under vacuum of < 5×10^-7^ Pa).

Atom probe specimens were prepared by focused ion beam following a standard recipe^6^ with final annular milling radius of 100 nm at 30 kV and 40 pA. Specimen cleaning was obtained by reducing the voltage of the focused ion beam to 5 kV and using a current of 40 pA for 30 to 60 seconds. Quantitative probing of the chemical composition at the nanometre scale was carried out with a CAMECA locale electrode atom probe (LEAP) 4000X HR in laser-assisted measurement mode. Specimens from thin films grown at *T** = 0.32 were evaporated with 30 pJ laser pulse energy, 250 kHz laser pulse frequency, 60 K base temperature, 0.5% detection rate and approximately 40 million ions were collected for the dataset shown in Fig. 4.

However, these parameters resulted in premature fracture of the specimens from thin films grown at *T** = 0.40 and the effect of the electric field is examined in Fig. SI8. Scanning electron and scanning transmission electron micrographs of a (Mg,Ca) solid solution (*T** = 0.40) were obtained directly after preparation (Fig. SI8a and b). Besides the (Mg,Ca) specimen of interest, another silicon specimen was located on the coupon (Fig. SI8c). Silicon is used as reference material in order to align the specimen position to the local electrode as well as the laser spot to the specimen position and this procedure is necessary every time the local electrode is changed. During the atom probe session, only this alignment step was done and therefore a voltage of approximately 3 kV was applied to the coupon, when the silicon reference was in front of the local electrode. Post-alignment observation of the (Mg,Ca) specimen of interest revealed that it appeared bent due to material accumulation even without intentional evaporation of this specimen (Fig. SI8d). It can be understood that too high electric field strengths result in premature fracture. Therefore, the laser pulse energy was increased from 30 to 100 pJ to reduce the electric field strength during evaporation. In order to reduce the measurement speed as well as the temperature of the specimen, the laser pulse frequency and the base temperature were also adjusted to 125 kHz and 30 K, respectively. Thereby, a successful measurement was performed and approximately 20 million ions were collected for the dataset shown in Fig. 5.

**Fig. SI8.** Damage of (Mg,Ca) solid solution specimens grown at *T** = 0.40. (**a**), Scanning electron microscopy (SEM) and (**b**), scanning transmission electron microscopy (STEM) of an atom probe specimen (*T** = 0.40) after preparation. (**c**), Overview of microtips on the silicon coupon. The (Mg,Ca) specimen of interest was positioned at a distance > 1 mm from a specimen used for laser alignment of the atom probe. (**d**), Appearance of (Mg,Ca) specimen after alignment procedure.

# References

^1^ Thornton, J. A. Influence of apparatus geometry and deposition conditions on the structure and topography of thick sputtered coatings. *J. Vac. Sci. Technol.* **11**, 666-670 (1974).

^2^ Keuter, P., Aghda, S. K., Music, D., Kümmerl, P. & Schneider J. M. Synthesis of Intermetallic (Mg_1-x_Al_x_)_2_Ca by Combinatorial Sputtering. *Materials* **12**, 3026 (2019).

^3^ Herbig, M. *et al.* Atomic-Scale Quantification of Grain Boundary Segregation in Nanocrystalline Material. *Phys. Rev. Lett.* **112**, 126103 (2014).

^4^ Kuzmina, M., Herbig, M., Ponge, D., Sandlöbes, S. & Raabe, D. Linear complexions: Confined chemical and structural states at dislocations. *Science* **349**, 1080-1083 (2015).

^5^ Diercks, D. R. *et al.* Three-dimensional quantification of composition and electrostatic potential at individual grain boundaries in doped ceria. *J. Mater. Chem. A* **4**, 5167-5175 (2016).

^6^ Thompson, K. *et al.* In situ site-specific specimen preparation for atom probe tomography. *Ultramicroscopy* **107**, 131-139 (2007).
